# Copy Number Alterations in Canine Urothelial Carcinomas: The Impact of Tumour Purity

**DOI:** 10.3390/vetsci13050459

**Published:** 2026-05-08

**Authors:** Marielle Appenzeller, Heike Aupperle-Lellbach, Alexandra Kehl, Robert Klopfleisch, Simone de Brot

**Affiliations:** 1 LABOKLIN GmbH & Co. KG, 97688 Bad Kissingen, Germany; kehl@laboklin.com; 2 School of Medicine, Institute of Pathology, Technical University of Munich, 80333 München, Germany; 3 Institute of Veterinary Pathology, Free University of Berlin, 14195 Berlin, Germany; robert.klopfleisch@fu-berlin.de; 4 Institute of Animal Pathology, COMPATH, University of Bern, 3012 Bern, Switzerland; simone.debrot@unibe.ch

**Keywords:** copy number alteration, comparative genomic hybridisation, molecular diagnostic, ddPCR, canine urothelial carcinoma, transitional cell carcinoma, dogs

## Abstract

Molecular genetic approaches have become indispensable in veterinary medicine for early tumour detection, confirming histomorphological diagnoses, and non-invasive diagnosis using urine samples. In canine urothelial carcinoma (UC), molecular testing includes *BRAF*^V595E^ mutation analysis and the identification of specific copy number alterations (CNAs). CNAs affect multiple genes simultaneously, leading to their overexpression or underexpression. Recurrent gene gains on canine chromosomes (CFA)13 and CFA36 and losses on CFA19 are highly prevalent in canine UCs. This study evaluates CNA test performance and the influence of tumour purity on CNA test results using 76 histologically confirmed UC tissue samples. The tumour region of each histopathological section was measured, and its proportion relative to the total tissue area was calculated. In total, 58/76 cases (76.3%) were CNA-positive, defined by a ratio greater than 1.23 for both CFA13/19 and CFA36/19. A total of 14/18 negative cases had a ratio > 1.23 for CFA13/19 or CFA36/19. The sensitivity of this CNA assay depends on the applied thresholds, with a tumour-to-total tissue ratio of ≥20% yielding positive CNA results in 84% of cases, rising to 89% at ≥40%. These findings indicate that <20% tumour content and a moderate to high proportion of copy number-neutral cells may be associated with reduced CNA detectability.

## Simple Summary

Molecular genetic diagnostics are increasingly important in veterinary medicine for early tumour detection, confirmation of histomorphological diagnoses, and non-invasive testing. In dogs with urothelial carcinoma (UC), testing not only for the *BRAF*^V595E^ mutation but also for copy number alterations (CNAs) can aid in diagnosis. CNAs can simultaneously influence multiple genes, leading to their overexpression or underexpression. In particular, gene gains in chromosomal regions on dog chromosomes (CFA)13 and CFA36, and gene losses in CFA19, are commonly found in canine UCs. This study examines CNA test performance and how tumour purity affects CNA test results in 76 confirmed UC tissue samples. The tumour region in each histopathological section was measured and calculated as a proportion of the total tissue area. Positive CNA results were found in 58/76 cases (76%), with ratios above 1.23 for both CFA13/19 and CFA36/19. In total, 14/18 negative cases had a ratio > 1.23 for CFA13/19 or CFA36/19. The CNA test detected tumours in 84% of samples with ≥20% tumour content, rising to 88% when the tumour comprised ≥ 40% of the tissue. These findings suggest that a tumour content below 20% and a higher proportion of copy number-neutral cells (e.g., muscle and inflammatory cells) can reduce CNA detectability.

## 1. Introduction

Urothelial carcinoma (UC), previously also known as transitional cell carcinoma, is one of the most common tumours of the urinary tract system [1]. UC comprises approximately 1.5–2% of all canine cancers and is highly malignant [1,2]. In dogs, a breed-specific predisposition to UC has been described, with Scottish Terriers, Shetland Sheepdogs, Beagles, Fox Terriers, and West Highland White Terriers being particularly affected [1,3,4].

UC is typically associated with nonspecific clinical signs, including pollakiuria, haematuria, stranguria, and tenesmus [2,5,6]. Consequently, these symptoms often mimic cystitis or urolithiasis, leading to delayed diagnosis and presentation at an advanced stage of the disease [1,2,3]. Thus, early detection of UC is important for therapeutic success, given that early-stage cancers typically respond more favourably to treatment [7,8]. Several diagnostic approaches are currently available for evaluating suspected UC, reflecting advances in cytological, imaging, and molecular techniques. While imaging techniques such as ultrasonography and radiography can detect urinary tract masses and abnormal growths, their ability to assess malignancy is limited [9,10,11]. Therefore, reliable differentiation between UC and benign lesions is challenging.

Cytological examination of urine sediment is a valuable diagnostic tool. However, its interpretation is limited if atypical transitional cells are absent. Inflammatory and reactive changes associated with bacterial cystitis may cause urothelial cell pleomorphism that mimics neoplasia, leading to potential misinterpretation [12,13,14]. Histopathological examination remains the gold standard for diagnosing UC, but it requires invasive surgical procedures, is expensive, and has been associated with the risk of tumour cell seeding [1,15]. In addition, biopsy sampling is frequently restricted by the anatomical inaccessibility of the tumour, often located in the urinary bladder trigone [3]—as well as patient-related factors such as size and sex, associated costs, and technical limitations within the clinic. Furthermore, the diagnostic utility of cytology and histology may be limited by sample quality [14,16,17].

To develop new diagnostic tools in veterinary oncology, research has increasingly focused on molecular genetic diagnostics [13,17,18]. Most canine UCs harbour a single base mutation in exon 15 of the *BRAF* gene, making *BRAF* mutation analysis a valid diagnostic option [17,19]. A breed predisposition for this *BRAF* mutation in UC has been reported in Scottish Terriers, Shetland Sheepdogs, and Beagles [4]. Mochizuki et al. developed an assay to detect the presence of the *BRAF*^V595E^ mutation in urine sediment cells [17]. The droplet digital polymerase chain reaction (ddPCR) assay to detect the canine *BRAF*^V595E^ mutation is remarkable for its sensitivity of 85% and specificity of >99% [17]. This mutation analysis enables a reliable diagnosis of *BRAF*-mutated UC. The high prevalence of the *BRAF* mutation in UC in dogs—reported to be between 67% [20] and 87% [19]—supports the use of *BRAF* mutation analysis as a valid diagnostic tool in routine diagnostics [4]. In contrast, the remaining UC cases lacking a *BRAF* mutation cannot be identified by this test.

Aneuploidy is a hallmark genetic alteration in human cancer, occurring in approximately 88% of malignancies, particularly in solid tumours [21]. Copy number alterations (CNAs) include both large-scale aneuploidy events and smaller, focal changes resulting from localised deletions or amplifications [22]. CNAs have been extensively characterised across tumour entities in human medicine. Certain human cancer types are predominantly driven by CNAs [23], including triple-negative pregnancy-associated breast cancer [24], serous ovarian carcinoma [25], and lung squamous cell carcinoma [26]. Moreover, other human neoplasms such as prostate cancer [27], colorectal cancer [28,29], lung adenocarcinomas [30], and meningiomas [31] exhibit recurrent CNA signatures that enable diagnostic, prognostic, and therapeutic classification. CNA burden, defined as the proportion of the tumour genome affected by copy number changes, serves as a fundamental measure of genomic instability [32]. Higher CNA burden is linked to poorer progression-free and overall survival. In comparison, lower CNA burden is associated with improved outcomes, highlighting its value as a measurable, clinically relevant prognostic factor [32].

Interestingly, analyses of canine UC biopsies have revealed aneuploidy across multiple canine chromosomes, classified as CNA [13]. CNAs can simultaneously involve multiple genes, leading to their overexpression or underexpression, and may affect segments of chromosomes or whole chromosomes [33]. High-frequency aneuploidy of canine chromosomes CFA13, 19, and 36 within a single cell represents a cytogenetic feature distinguishing canine UC from benign urothelium [13]. Shapiro et al. developed a fluorescence in situ hybridisation (FISH) assay for the detection of UC in biopsies and in urine. CFA13 and CFA36 gains and losses in CFA19 were observed in 97%, 84%, and 77% of the UC cohort, respectively [13]. Furthermore, the presence of two or more of these three aberrations in cells from a urinary tract specimen demonstrates extremely high (>99%) sensitivity and specificity for the detection of neoplasia [13]. In another study, a multiplexed ddPCR assay was developed for the detection and quantification of CNAs in defined regions of these canine chromosomes for diagnostic purposes in canine UC [34].

In the study of Mochizuki et al., the CNA ddPCR assay in canine UC tissue specimens demonstrated 100% sensitivity and specificity for distinguishing UC from benign urothelium, with no false positives among 25 control specimens [34]. In human bladder cancer, Li et al. also developed a ddPCR assay for detecting fibroblast growth factor receptor substrate 2 (FRS2) copy number in UC bladder samples with 100% sensitivity and specificity [35]. The histopathological description of the UC cases analysed in the study of Mochizuki et al. was based on the tumour staging and the presence or absence of muscular invasion [34]. It is already known that, in cases with muscle involvement, both the frequency and size of these CNAs increase [13]. Mochizuki et al. provided no information regarding the purity or quality of the tumour section from which the paraffin-embedded material used for ddPCR analysis was obtained [34]. Due to the relatively small sample cohort (UC, *n* = 31; non-neoplastic bladder specimens, *n* = 25) and the likely pre-selection of UC tissue samples based on high tumour purity and minimal proportions of non-neoplastic or other pathological tissue, the assay was primarily evaluated for its practicality [34]. In clinical practice, the sample material is considerably more heterogeneous, which is why a test sensitivity of 100% cannot be reliably achieved.

In contrast, in urine samples, neoplasia was not detected by CNA in 33% of the 18 cases [34]. This limitation was attributed to an excess of copy number-neutral inflammatory cells in the urine secondary to bacterial infections, thereby increasing the risk of false-negative findings. In contrast, red blood cells do not affect copy number assessment because they lack genomic DNA. In conclusion, a key requirement for a reliable analysis is the presence of a sufficient proportion of neoplastic cells in the sample. Furthermore, high sample quality is essential, and microbial contamination or bacterial growth should be kept to a minimum. In samples containing non-urothelial components (e.g., leucocytes), UC-specific CNA may not be detectable due to dilution or interference from non-neoplastic DNA [34].

In summary, CNA analysis represents a valid and promising alternative approach for detecting *BRAF* wildtype UCs. However, analysis of the *BRAF* mutation should be clearly distinguished from CNA analysis, as it is a distinct molecular assay. Routine diagnostic laboratories often offer these two tests either concurrently or sequentially for the assessment of UC, as both analyses use the same sample material (isolated DNA). In Europe, this additional test is offered by LABOKLIN GmbH & Co. KG under the name “BRAF Complete”, while in the United States, it is provided by Antech as “CADET^®^ BRAF-PLUS” (Fountain Valley, CA, USA).

One current limitation of this CNA-based assay for UC diagnosis is its inability to achieve 100% sensitivity in routine tissue samples, due to suboptimal tumour purity and heterogeneous tissue composition. At the same time, specificity remains at 100% for distinguishing UC from benign urothelium. This study investigates potential causes of CNA-negative results in histologically confirmed UC tissue samples, focusing on histopathological factors, such as tumour cell proportion, that influence assay performance. This study aims to evaluate the capabilities and limitations of this assay for CNA detection with respect to histopathological features, specifically tumour content and tumour morphology, thereby contributing to the further optimisation of a reliable molecular genetic diagnostic tool. This work represents an important translational step from method development towards realistic diagnostic implementation, thereby providing novel insights into their clinical relevance and addressing an important gap in the growing role of molecular methods in veterinary oncology.

## 2. Materials and Methods

A dataset comprising 76 diagnostic canine UC samples submitted for histopathological examination to the Institute of Animal Pathology, University of Bern (Switzerland), and the School of Veterinary Medicine and Science, University of Nottingham (UK), as well as 24 diagnostic canine non-neoplastic bladder samples submitted to LABOKLIN GmbH & Co. KG (Germany), was included in this study. The samples were originally submitted for routine diagnostic purposes, not specifically for scientific research. In this study, no cases were excluded due to suboptimal sample quality. This approach was intentionally chosen to better reflect routine diagnostic conditions, in which samples of variable, and sometimes limited, quality are frequently submitted. Breed designations used in this study followed the nomenclature of the Fédération Cynologique Internationale (FCI). Dogs belonging to breeds not officially recognised by the FCI were grouped as crossbreed dogs.

Inclusion criteria for the UC samples were (1) histopathologically confirmed UC and (2) a CNA test analysis result. Inclusion criteria for the non-neoplastic samples used as a control group were (1) histopathologically confirmed cystitis (*n* = 16) or bladder polyp (*n* = 8), and (2) a CNA analysis test result.

All 76 UC samples and 24 non-neoplastic samples consisted exclusively of formalin-fixed, paraffin-embedded (FFPE) tissue. Haematoxylin and eosin-stained tissue sections of the UC cases were digitised (scanner: 3DHISTECH Pannoramic 250 Flash III, 3DHISTECH Kft., Budapest, Hungary) and assessed using the 3DHISTECH Slide Viewer version 2.7 software by a board-certified veterinary pathologist (S.d.B.), and were histopathologically confirmed as UC. Growth patterns were assigned to each sample: conventional urothelial, solid, cribriform, cystic, and mixed.

**Figure 1:**
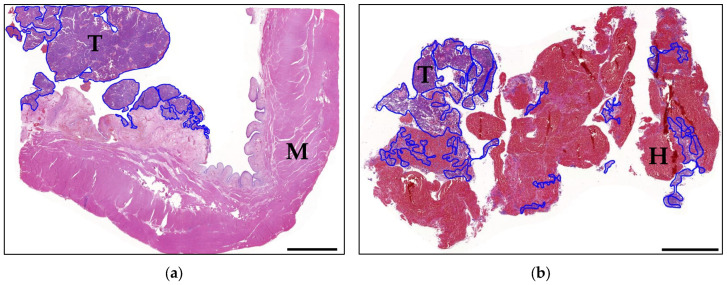
Overview images of the histopathological examination and the manually segmented tumour regions (outlined in blue). (**a**) Transmural sample from a UC of a 12-year-old male neutered West Highland White Terrier (case 76). The biopsy contains a large proportion of bladder wall muscle. HE stain; bar = 5 mm. (**b**) Small UC biopsies of a 12-year-old female neutered Border Collie with minimal neoplastic tissue and marked haemorrhage (case 5). HE stain; bar = 1 mm. UC, urothelial carcinoma; HE, haematoxylin and eosin; T, tumour; M, muscle; H, haemorrhage.

The entire tissue area of each tissue section was automatically detected, and the corresponding tumour region was manually segmented (Figure 1). Tumour segmentation was performed by a single pathologist and confirmed by the supervisors (S.d.B. and H.A.-L.) on a subset of cases. Non-tumour areas, such as haemorrhagic spaces, were included in the total tissue area for analysis. This enabled the calculation of the tumour fraction for each case, defined as the proportion of tumour area relative to the total tissue area. In the following, this value is referred to as the “tumour-to-total tissue ratio”.

As part of the initial molecular genetic analysis, *BRAF* mutation testing was performed to detect the c.1784T>A substitution. DNA was isolated from two 10 µm thick FFPE tissue sections and subsequently used for the *BRAF* mutation analysis using ddPCR in accordance with the c*BRAF*^V595E^ ddPCR protocol established and described by Mochizuki et al. [17].

The existing DNA, used for *BRAF* mutation testing, was also used to perform the CNA assay as described by Mochizuki et al. [34]. The ratios of CFA13/19 and CFA36/19 were calculated for each sample and subsequently normalised using a correction factor based on the positive control included in each ddPCR run. Samples were classified as CNA-positive if both normalised ratios (CFA13/19 and CFA36/19) exceeded the predefined ratio × correction factor threshold of 1.23, as established by Mochizuki et al. [34].

The ddPCR was performed on the QX200 Droplet Digital System (Bio-Rad, Hercules, CA, USA) using ddPCR Supermix for Probes (Bio-Rad, Hercules, CA, USA) and a thermal cycler (Biometra TOne, Jena, Germany). For ddPCR analysis, assays were considered valid only if at least 5000 total droplet events per reaction were achieved and at least 100 positive droplets were detected for each target (CFA13, CFA19, and CFA36). In addition, both the no-template control and positive control were required to meet predefined quality criteria.

Statistical analyses were performed using Microsoft Excel 365 and IBM SPSS Statistics, version 32.0.0.0 (134). Group comparisons were conducted using the non-parametric Mann–Whitney U test. Univariable and multivariable logistic regression analyses were performed to assess the association between tumour tissue proportion and CNA positivity. In the multivariable model, additional histopathological variables, including the proportion of inflammation, muscle tissue, necrosis, and haemorrhage, were included as covariates. Odds ratios (ORs) with 95% confidence intervals (CIs) were calculated, and a *p*-value < 0.05 was considered statistically significant.

To assess the influence of tumour-to-total tissue ratio on CNA test performance, tumour proportions were visually inspected using a boxplot of all UC cases. Based on this distribution, a series of pragmatic tumour content thresholds (10%, 20%, and 40%) was defined to stratify the cases. For each tumour fraction threshold, sensitivity was defined as the proportion of CNA-positive cases among all UC samples at or above the respective threshold, which were included in the denominator. Non-neoplastic bladder samples were included to assess specificity.

## 3. Results

### 3.1. Sample Population

Formalin-fixed tissue samples from 76 dogs with histopathologically confirmed UC were included in this study. The signalment data of these dogs are provided in Table S1.

Across all cases, a predominance of female dogs was observed (47 females and 29 males). The majority of dogs were neutered (*n* = 61); only two males and one female were intact at the time of sampling. For the remaining 12 dogs, neutering status was unavailable. The median age at diagnosis was 10 years (range 6–14).

In addition to the crossbreed dogs (*n* = 17), the most frequently represented breeds were Labrador Retrievers (*n* = 10), West Highland White Terriers (*n* = 6), Cavalier King Charles Spaniels (*n* = 5), and Jack Russell Terriers (*n* = 5). Overall, terrier breeds accounted for a noticeable proportion of cases, comprising 18 dogs from six different terrier breeds, including West Highland White Terriers (*n* = 6), Jack Russell Terriers (*n* = 5), Scottish Terriers (*n* = 3), Fox Terriers (*n* = 2), Staffordshire Bull Terriers (*n* = 1), and Tibetan Terriers (*n* = 1). Altogether, 67 out of 76 dogs (88%) tested positive for the canine V595E mutation on the *BRAF* gene. All terrier samples and all three Shetland Sheepdogs included in the cohort had a *BRAF*-mutated UC.

The CNA-negative control group comprised 24 non-neoplastic bladder samples, including 16 cases of cystitis and 8 bladder polyps, all derived from FFPE specimens. The cohort consisted of 10 females and 13 males; sex was not reported for one case. Age information was available for 23 dogs, with a median age at diagnosis of 8 years (range 1–12). All of these control cases tested negative for the *BRAF* mutation.

### 3.2. Histopathological Examination

#### Tumour Morphology

**Figure 2:**
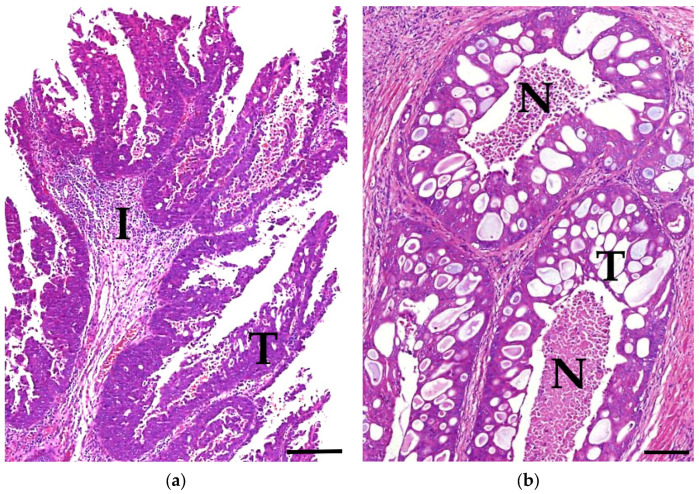
Histopathological growth patterns of urothelial carcinomas (UC); examples: (**a**) UC (T) with conventional urothelial growth pattern and pronounced mixed cellular inflammation (I) of a 13-year-old female neutered crossbreed dog; case 30; HE stain; bar = 0.2 mm. (**b**) UC (T) with a glandular/nested growth pattern and severe comedonecrosis (N) of a 13-year-old male neutered crossbreed dog; case 31; HE stain; bar = 0.1 mm. UC, urothelial carcinoma; T, tumour; HE, haematoxylin and eosin.

Histopathologically, the carcinomas most commonly showed a conventional urothelial growth pattern (*n* = 37; 49%; Figure 2a). Furthermore, solid (*n* = 3; 4%), cribriform (*n* = 2; 3%), glandular/nested (*n* = 2; 3%; Figure 2b), microinvasive (*n* = 2; 3%), and cystic (*n* = 1; 1%) growth patterns, as well as squamous differentiation (*n* = 1; 1%) and mixed forms (*n* = 28; 37%), were present. Of the 76 samples analysed, seven showed reduced or poor tissue preservation quality.

**Figure 3:**
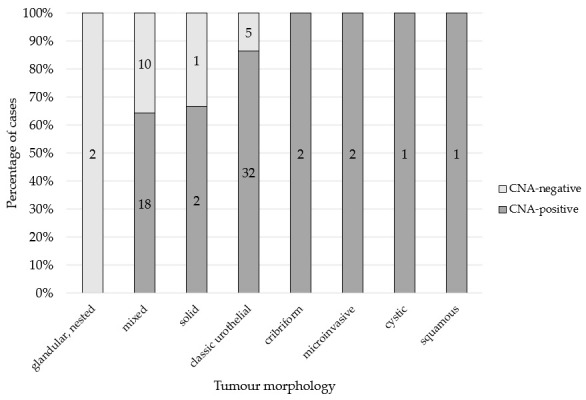
The 100% stacked bar chart of the histopathological evaluation of the 76 UC samples, categorised according to their tumour morphology. Numbers within the bars represent case counts per tumour morphology group. Samples are stratified by CNA test result (positive vs. negative). UC, urothelial carcinoma; CNA, copy number alteration.

When evaluating the eight groups of distinct growth patterns with respect to their CNA results, negative test outcomes were observed in only four groups (Figure 3). Among all CNA-negative cases in the cohort (*n* = 18), negative results were identified exclusively in tumours with glandular/nested (2/18; 11%), conventional urothelial (5/18; 28%), solid (1/18; 6%), and mixed growth patterns (10/18; 56%). Within the conventional urothelial growth pattern group (*n* = 37), 5 cases (14%) were CNA-negative. In the solid growth pattern group (*n* = 3), one case (33%) yielded a negative CNA result. Both cases with a glandular/nested growth pattern (cases 53 and 66) had a negative CNA result. Notably, their tumour-to-total tissue ratios were 8% and 11%, indicating that only a minimal proportion of neoplastic tissue was present in the respective histopathological sections. Of the 28 cases with a mixed growth pattern, 10 (36%) were not detected as UC by CNA analysis. All six cases exhibiting cribriform, microinvasive, cystic, or squamous growth patterns were correctly identified as UC. However, the effect of morphology could not be statistically assessed due to small group sizes.

### 3.3. CNA Analysis

**Figure 4:**
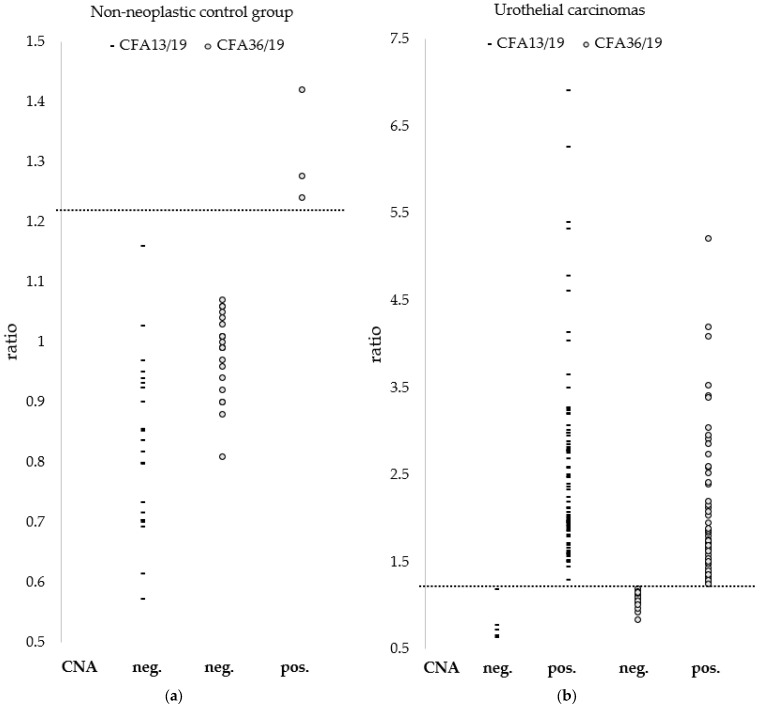
Copy number ratios of (**a**) the 24 non-neoplastic samples and (**b**) the 76 UC samples, stratified by CFA13/19 and CFA36/19, and categorised according to CNA status (positive vs. negative). Dashed lines indicate the threshold value of 1.23 for both CFA13/19 and CFA36/19. A case was classified as CNA-positive only when both ratios exceeded 1.23, as defined by Mochizuki et al. [34], distinguishing normal from aberrant copy number ratios. UC, urothelial carcinoma; CFA, canine chromosome; CNA, copy number alteration; neg., negative; pos., positive. Adapted from Mochizuki et al. [34].

All 24 samples of cystitis and polyps yielded negative CNA results (Figure 4a). Three cystitis samples (cases 90, 97, and 98) exhibited a CFA36/19 ratio exceeding the threshold of 1.23 as defined by Mochizuki et al. [34] (1.24, 1.28, and 1.42); however, their corresponding CFA13/19 ratios remained clearly below this threshold (0.80, 1.16, and 0.85).

In the UC cohort, the ratios of CFA13/19 ranged from 0.63 to 6.91, whereas those of CFA36/19 ranged from 0.84 to 5.21 (Figure 4b). A total of 58 out of 76 cases (76%) demonstrated a positive CNA result, defined by a ratio greater than 1.23 for both CFA13/19 and CFA36/19.

Three *BRAF* wildtype UCs were successfully classified as neoplasia based on CNA testing: (1) Case 1 is a UC with a mixed growth pattern with severe haemorrhage of a 12-year-old male neutered American Cocker Spaniel with a tumour-to-total tissue ratio of 58%. (2) Case 17 is a mixed UC with significant inflammation of an 8-year-old female neutered crossbreed dog. The tumour-to-total tissue ratio was measured at 10%. (3) Case 70 is a UC with a conventional urothelial growth pattern and a high tumour purity (75%) of an 8-year-old female neutered Welsh Springer Spaniel.

In 18 cases, the threshold (>1.23) for both ratios, CFA13/19 and CFA36/19, was not exceeded; therefore, these cases were considered CNA-negative. In total, 14/18 negative cases had a ratio > 1.23 in one of these ratios (CFA13/19, *n* = 13; CFA36/19, *n* = 1). Among these, all 14 cases harboured a *BRAF* mutation, whereas four cases were not identified as UC by both molecular diagnostic analyses.

**Figure 5:**
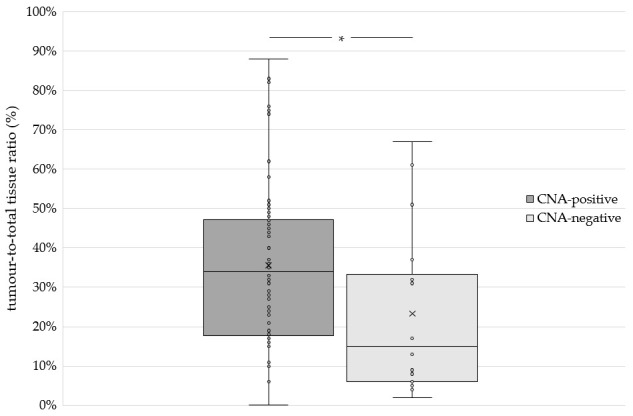
Boxplot illustrating the distribution of tumour content (%) in histological samples from 76 UC cases stratified by CNA test result (positive vs. negative). * *p* = 0.02. UC, urothelial carcinoma; CNA, copy number alteration.

The tumour-to-total tissue ratio, relative to the CNA test results, for the 76 histological samples is shown in Figure 5. In the 18 CNA-negative samples, the tumour-to-total tissue ratio ranged from 2% to 67% (median = 14.9%), whereas CNA-positive samples generally exhibited higher ratios (median = 33.7%; range = 0.1–88%). The tumour-to-total tissue ratio differed significantly between CNA-positive and CNA-negative cases (median 34% vs. 15%, Mann–Whitney U test, *p* = 0.02). Logistic regression analysis revealed that samples with a tumour tissue proportion ≥ 20% had significantly higher odds of yielding a positive CNA result compared to samples with <20% tumour content (OR = 3.281, 95% CI: 1.099–9.794; *p* = 0.03). In an additional multivariable logistic regression analysis including histopathological factors such as inflammation, muscle tissue, necrosis, and haemorrhage, samples with higher proportions of non-neoplastic tissue components tended to show lower odds of a positive CNA result compared to samples with higher tumour purity and absence of these non-tumour components (OR = 0.157, 95% CI: 0.018–1.392). However, this association did not reach statistical significance (*p* = 0.096).

Visual inspection of the boxplot (Figure 5) suggests that the majority of CNA-positive cases had tumour proportions above approximately 20%, although exceptions were observed. To assess the influence of tumour-to-total tissue ratio on CNA test performance, sensitivity and specificity were evaluated at multiple tumour proportion thresholds (Table 1). This overview of sensitivity by threshold demonstrates that a tumour-to-total tissue ratio of 20% in the sample is sufficient to yield a positive CNA result in 84% of cases. Increasing the tumour fraction to 40% raises this proportion to 88%. Notably, the CNA test achieved 100% specificity across all tumour fraction thresholds in non-neoplastic control samples, including cases of confirmed cystitis (*n* = 16) and bladder polyps (*n* = 8), which consistently yielded CNA-negative results.

A total of 16 cases of the UC cohort exhibited a tumour-to-total tissue ratio of <20% (median = 15%) yet still yielded a positive CNA result. Among these cases were five crossbreed dogs, three Labrador Retrievers, two Cavalier King Charles Spaniels, two Shetland Sheepdogs, and one each of Cocker Spaniel, Jack Russell Terrier, Pointer, and Weimaraner. Of these dogs, 10 were female and 6 male, with a median age at diagnosis of 9 years (range 7–13). The non-tumour components in these cases consisted of a high proportion of muscle tissue (*n* = 5), inflammation (*n* = 4), a combination of muscle and inflammation (*n* = 3), necrosis (*n* = 3), and haemorrhage (*n* = 1). The ratios of CFA13/19 ranged from 1.44 to 3.26, whereas the ratios of CFA36/19 ranged from 1.27 to 5.21. In case 47, a tumour-to-total tissue ratio as low as 0.1% was sufficient to isolate enough DNA to detect a relevant CNA.

In eight cases, a tumour-to-total tissue ratio exceeding 20% (median = 34.5%) was identified, yet the CNA result remained negative overall. All of these cases tested positive for the *BRAF* mutation. The cohort included three Labrador Retrievers, two Cocker Spaniels, and one each of Cavalier King Charles Spaniel, crossbreed dog, and Shetland Sheepdog. The group comprised five female and three male dogs, with a median age at diagnosis of 11.5 years (range 7–14). In these cases, the non-tumour components were predominantly inflammation (*n* = 2), muscle tissue (*n* = 1), and necrosis (*n* = 1). In 7/8 cases, a CFA13/19 ratio > 1.23 was observed (range 1.30–4.61), while the CFA36/19 ratio ranged from 0.84 to 1.10. In the remaining case (case 55), a 13-year-old neutered female Labrador Retriever, the CFA13/19 ratio reached 1.19 and the CFA36/19 ratio 2.20. This UC, with a mixed growth pattern of conventional urothelial, solid, and cystic components and marked mixed cellular inflammation, exhibited a tumour-to-total tissue ratio of 31%. In cases 8 and 15, the tumour-to-total tissue ratios were 37% and 51%, respectively; no non-tumour components were present except for haemorrhage, yet both yielded negative CNA results.

## 4. Discussion

This study aimed to address the existing gap in understanding the impact of tumour tissue proportion on histopathological samples for reliable CNA analysis. To this end, a dataset of 76 diagnostic samples, all histopathologically confirmed as UC, was analysed. Scottish Terriers, terrier breeds in general, and Shetland Sheepdogs—previously reported to be predisposed to UC, particularly *BRAF*-mutated UC—were also overrepresented in our study population. All cases from these predisposed breeds tested positive for the *BRAF* mutation. Our study cohort, therefore, appears to be representative, as the breed distribution reflects the known epidemiological pattern of UC in the population.

Consistent with Mochizuki et al. [34], we demonstrated that in tumours with predominantly moderate to high purity, CNA analysis reliably yielded positive results, detecting UC with 100% specificity and 84–88% sensitivity. The adopted threshold of 1.23 for both ratios (CFA13/19 and CFA36/19), as proposed by Mochizuki et al. [34], may not be fully optimised for heterogeneous clinical tissue specimens, in which variable proportions of non-neoplastic components are present. This is further illustrated by our study, which found that 14/18 CNA-negative cases exhibited a ratio > 1.23 in at least one of the two markers, suggesting that the requirement for both ratios to exceed the threshold for defining a CNA-positive result may reduce assay sensitivity in more heterogeneous samples. In this context, alternative interpretative strategies, such as considering a single-ratio exceedance or implementing a defined grey zone, may merit further evaluation in future validation studies. However, in the present study, we adhered strictly to the originally published protocol to ensure methodological consistency and comparability with previous work [34].

Only cases with high tumour purity were likely included in the study of Mochizuki et al. [34]. The tumours were classified according to their invasiveness into the different layers of the bladder: the lamina propria, muscularis propria, and perivesical tissues. However, the study of Mochizuki et al. did not report the proportion of haemorrhage, necrosis, or inflammation, nor indicate whether any cases contained such non-tumour components and to what extent [34]. The reported 100% sensitivity and specificity of this CNA assay in tissue samples could not be replicated in urine samples due to copy number-neutral inflammatory cells in the specimens of this study—the CNA testing failed in 33% of urine DNA samples [34]. The authors suggested that a secondary bacterial infection caused this inflammation [34].

The 76 histopathologically confirmed UC cases in our study were examined at multiple levels. Specifically, tumour morphology and tumour purity, defined as the tumour-to-total tissue ratio, were systematically assessed. Both glandular/nested cases (cases 53 and 66) showed CNA negativity and very low tumour fractions (8% and 11%, respectively), indicating that CNA detectability in this dataset appears to be driven primarily by tumour content rather than morphology per se. Among all 76 UC cases, 58 (76%) were correctly identified by CNA analysis. In the 18 CNA-negative cases, the tumour-to-total tissue ratio was very low, no CNAs were present across the CFAs, or the few alterations present effectively cancelled each other out in the CFA ratios.

The subsequent CNA analysis in our study yielded two major findings. First, a minimum proportion of pure tumour tissue is required for CNA analysis to produce true-positive results. Second, this minimum threshold is strongly influenced by the composition of the remaining tissue within the sample. Therefore, only a general recommendation can be made for CNA testing as a UC marker in routine diagnostics, as test sensitivity critically depends on the proportion of non-tumour tissue. Many nucleated non-tumour cells, such as inflammatory or muscle cells, are copy number-neutral. Consequently, the proportion of DNA derived from these copy-neutral cells may affect the 13/19 and 36/19 ratios used in CNA analysis, thereby shifting the signal toward a neutral profile.

Multiple factors influence CNA analysis. The proportion of non-neoplastic cells contributes to the overall CNA result to a certain extent. When tumour cells harbour high copy numbers of CFA13 and CFA36, the presence of non-neoplastic cells—characterised by normal CFA13/19 and CFA36/19 ratios—has only a limited impact on the final CNA outcome. In contrast, if tumour cells exhibit only low-level CNA of CFA13 and CFA36, the influence of non-neoplastic cells within the examined tissue becomes more pronounced, shifting the ratios toward lower values. This effect may obscure UC and lead to a false-negative CNA finding. In human bladder cancer, a whole-genome sequencing study also demonstrated that FRS2 copy number can increase by 3- to 25-fold in tumour tissues [36], and this amplification is associated with poor prognosis, suggesting that it acts as a driver alteration [37]. However, the exact number of CFA copies present per cell remains unknown and can currently be considered a “black box”.

Advances in molecular pathology have led to its increasing integration into routine clinical diagnostics, where it serves as a complementary approach to conventional morphological pathology and facilitates the identification of genetic homologies across species. Accordingly, a study examining the progression from human colorectal adenoma to carcinoma found a higher number of chromosomal aberrations in carcinomas than in adenomas [28]. These alterations appear to play a key role in the progression from adenoma to carcinoma [28]. A comparative analysis of canine and human colorectal cancer (CRC) revealed a high degree of genetic homology [29]. Canine CRCs showed recurrent CNAs affecting orthologs of human CRC genes, with total CNA burden correlating with tumour stage. Shared CNAs between species tended to involve evolutionarily stable regions, suggesting that these alterations are more likely to be cancer-causative, while species-specific CNAs localised to unstable genomic regions.

Our findings can be transferred to routine diagnostics. It is important to consider the influence of tissue composition on the reliability of CNA analysis. In tissue samples, the presence of muscle cells, which are part of the population of copy number-neutral cells, can affect the accuracy of CNA results by diluting tumour-derived DNA. Clinically, human bladder cancer is stratified into non-muscle-invasive (NMIBC) and muscle-invasive (MIBC) subtypes, with CNAs detected in over 20% of NMIBC and up to 30% of MIBC cases [38]. In contrast, the presence of muscle cells is unlikely to affect urine-based analyses, as they are not shed into the urine. Inflammatory cells, however, may be present in urine in cases of cystitis and can contribute copy number-neutral DNA to the analysed sample, thereby potentially influencing CNA results. For this reason, urine samples intended for CNA analysis must undergo prior cytological examination. Such evaluation allows the assessment of the cellular composition of the sample and may also enable the detection and preliminary evaluation of exfoliated epithelial cells.

In veterinary medicine, insights into the prognostic value of CNA analyses are still limited. In human medicine, however, CNA analyses are used not only for diagnostic purposes but also to provide prognostic information that can be applied at the molecular genetic level. In human meningiomas, molecularly integrated grading approaches that combine histopathology with CNAs have been shown to improve the prediction of tumour recurrence [31]. Inclusion of recurrent CNAs enables more accurate risk stratification and reclassification of histologically benign tumours with higher genetic risk, providing robust and clinically applicable prognostic markers beyond WHO grading alone [31]. Wang et al. show that CNAs in human prostate cancer are strongly associated with metastasis and patient outcomes [27]. Specific CNA signatures in prostate cancer are strongly linked to metastatic disease, poor overall survival (OS), and poor progression-free survival (PFS), whereas others are associated with improved OS and improved PFS [27].

The CNA burden of a tumour also represents an important prognostic factor. Our study is limited in its assessment of CNA burden, highlighting the need for further research investigating the frequency of CNAs in canine UC specimens and their potential prognostic relevance. In contrast, high-grade human serous ovarian carcinoma is among the most aneuploid cancer types, exhibiting a high CNA burden in which up to two-thirds of genes in the primary tumour may be affected by CNA [25]. In triple-negative pregnancy-associated breast cancer in humans, high CNA burdens and recurrent losses and gains in chromosomes are associated with tumour progression [24].

Another limitation of this study is the relatively low proportion of *BRAF*-negative cases in our cohort. However, a subset (3/7; 43%) of these *BRAF*-negative cases could be identified through CNA analysis, highlighting the complementary diagnostic value of this approach. Other potentially relevant factors, including tissue fixation time, paraffin quality, and FFPE block storage conditions, were not recorded systematically and could therefore not be considered or standardised in this study. In addition, although predefined ddPCR quality criteria (including minimum droplet counts and positive droplet thresholds) were applied, variability in pre-analytical FFPE-related DNA quality may still have influenced assay performance and potentially contributed to false-negative results.

We applied the ddPCR assay described by Mochizuki et al. [34] to a larger cohort of routine canine UC specimens, confirming previous findings and highlighting additional limitations of this CNA analysis. In human medicine, several studies have also shown that ddPCR can reliably detect specific CNAs in cancer. In oral squamous cell carcinomas [39] and melanocytic neoplasms [40], ddPCR-based assays showed good concordance with array methods in identifying recurrent gains and losses. The ddPCR assays have clinical value in differentiating between benign oral lesions and those that are at risk of progressing to oral cancer [39] and aid in the diagnosis of histopathologically ambiguous melanocytic tumours [40].

By demonstrating the utility of ddPCR assays in both human [35] and canine [34] UC samples, these results underscore the translational value of CNA-based diagnostics across species. Furthermore, these findings highlight ddPCR as a rapid, sensitive, and cost-effective alternative to conventional immunohistochemistry and FISH testing in tissue biopsies. In addition, they support the broader potential of CNA profiling, suggesting that comparable approaches may be implemented in veterinary oncology as CNA landscapes across tumour entities are increasingly defined.

## 5. Conclusions

This study demonstrates that CNA analysis of UC tissue is influenced by multiple factors, including tumour morphology, the number of copy number-neutral cells, and the number of CFA copies per cell, with tumour purity playing a central role. A tumour-to-total tissue ratio of 20% is sufficient to achieve positive CNA results in 84% of cases, increasing to 88% at 40% tumour content. The presence of non-neoplastic cells in the sample can reduce CNA detectability, particularly when the number of relevant CFA copies per cell is low.

## Figures and Tables

**Table 1:** Sensitivity of CNA testing across varying tumour-to-total tissue thresholds.

Tumour-to-Total Tissue Threshold	*n* Total	*n* CNA-Positive	*n* CNA-Negative	Sensitivity
-	76	58	18	76%
≥10%	67	56	11	84%
≥20%	50	42	8	84%
≥40%	26	23	3	88%

## Data Availability

The original contributions presented in the study are included in the article/Supplementary Materials; further inquiries can be directed to the corresponding authors.

## Supplementary Materials

The following supporting information can be downloaded at: https://www.mdpi.com/article/10.3390/vetsci13050459/s1, Table S1: Signalment data for each of the 76 canine urothelial carcinomas and the 24 non-neoplastic bladder samples, detailing breed, sex, neutering status, age at the time of sampling, *BRAF*^V595E^ mutation test results, CNA results, tissue and tumour measurements, and tumour morphology.

## Abbreviations

The following abbreviations are used in this manuscript:

ddPCR	Droplet digital polymerase chain reaction
CI	Confidence interval
CNA	Copy number alteration
CRC	Colorectal cancer
FCI	Fédération Cynologique Internationale
FRS2	Fibroblast growth factor receptor substrate 2
OR	Odds ratio
OS	Overall survival
*p*	*p*-value
PFS	Progression-free survival
UC	Urothelial carcinoma

## References

[1] Mutsaers A.J., Widmer W.R., Knapp D.W. (2003). Canine Transitional Cell Carcinoma. J. Vet. Int. Med..

[2] Norris A.M., Laing E.J., Valli V.E., Withrow S.J., Macy D.W., Ogilvie G.K., Tomlinson J., McCaw D., Pidgeon G., Jacobs R.M. (1992). Canine bladder and urethral tumors: A retrospective study of 115 cases (1980–1985). J. Vet. Int. Med..

[3] Knapp D.W., Glickman N.W., DeNicola D.B., Bonney P.L., Lin T.L., Glickman L.T. (2000). Naturally-occurring canine transitional cell carcinoma of the urinary bladder A relevant model of human invasive bladder cancer. Urol. Oncol..

[4] Appenzeller M., Kehl A., Törner K., Jensen K.C., Klopfleisch R., Aupperle-Lellbach H. (2025). BRAF Mutation Analysis: A Retrospective Evaluation of 8365 Diagnostic Samples with a Special View on Canine Breeds (2018–2024). Vet. Sci..

[5] Henry C.J. (2003). Management of transitional cell carcinoma. Vet. Clin. North Am. Small Anim. Pract..

[6] Bryan J.N., Henry C.J., Turnquist S.E., Tyler J.W., Liptak J.M., Rizzo S.A., Sfiligoi G., Steinberg S.J., Smith A.N., Jackson T. (2006). Primary Renal Neoplasia of Dogs. J. Vet. Int. Med..

[7] Loomans-Kropp H.A., Umar A. (2019). Cancer prevention and screening: The next step in the era of precision medicine. npj Precis. Oncol..

[8] Dhawan D., Ramos-Vara J.A., Utturkar S.M., Ruple A., Tersey S.A., Nelson J.B., Cooper B.R., Heng H.G., Ostrander E.A., Parker H.G. (2022). Identification of a naturally-occurring canine model for early detection and intervention research in high grade urothelial carcinoma. Front. Oncol..

[9] Hanazono K., Fukumoto S., Endo Y., Ueno H., Kadosawa T., Uchide T. (2014). Ultrasonographic findings related to prognosis in canine transitional cell carcinoma. Vet. Radiol. Ultrasound.

[10] Macrì F., Di Pietro S., Mangano C., Pugliese M., Mazzullo G., Iannelli N.M., Angileri V., Morabito S., de Majo M. (2018). Quantitative evaluation of canine urinary bladder transitional cell carcinoma using contrast-enhanced ultrasonography. BMC Vet. Res..

[11] Heng H.G., Ramos-Vara J.A., Fulkerson C.M., Fourez L.M., Knapp D.W. (2022). Ultrasonographic detection of apex nodules in the urinary bladder of Scottish Terriers. Vet. Radiol. Ultrasound.

[12] Straub J., Strittmatter F., Karl A., Stief C.G., Tritschler S. (2013). Ureterorenoscopic biopsy and urinary cytology according to the 2004 WHO classification underestimate tumor grading in upper urinary tract urothelial carcinoma. Urol. Oncol..

[13] Shapiro S.G., Raghunath S., Williams C., Motsinger-Reif A.A., Cullen J.M., Liu T., Albertson D., Ruvolo M., Bergstrom Lucas A., Jin J. (2015). Canine urothelial carcinoma: Genomically aberrant and comparatively relevant. Chromosome Res..

[14] Gentilini F., Palgrave C.J., Neta M., Tornago R., Furlanello T., McKay J.S., Sacchini F., Turba M.E. (2022). Validation of a Liquid Biopsy Protocol for Canine BRAFV595E Variant Detection in Dog Urine and Its Evaluation as a Diagnostic Test Complementary to Cytology. Front. Vet. Sci..

[15] Anderson W.I., Dunham B.M., King J.M., Scott D.W. (1989). Presumptive subcutaneous surgical transplantation of a urinary bladder transitional cell carcinoma in a dog. Cornell Vet..

[16] Knapp D.W., Ramos-Vara J.A., Moore G.E., Dhawan D., Bonney P.L., Young K.E. (2014). Urinary bladder cancer in dogs, a naturally occurring model for cancer biology and drug development. ILAR J..

[17] Mochizuki H., Shapiro S.G., Breen M. (2015). Detection of BRAF Mutation in Urine DNA as a Molecular Diagnostic for Canine Urothelial and Prostatic Carcinoma. PLoS ONE.

[18] Thomas R., Wiley C.A., Droste E.L., Robertson J., Inman B.A., Breen M. (2023). Whole exome sequencing analysis of canine urothelial carcinomas without BRAF V595E mutation: Short in-frame deletions in BRAF and MAP2K1 suggest alternative mechanisms for MAPK pathway disruption. PLoS Genet..

[19] Decker B., Parker H.G., Dhawan D., Kwon E.M., Karlins E., Davis B.W., Ramos-Vara J.A., Bonney P.L., McNiel E.A., Knapp D.W. (2015). Homologous Mutation to Human BRAF V600E Is Common in Naturally Occurring Canine Bladder Cancer--Evidence for a Relevant Model System and Urine-Based Diagnostic Test. Mol. Cancer Res..

[20] Mochizuki H., Kennedy K., Shapiro S.G., Breen M. (2015). BRAF Mutations in Canine Cancers. PLoS ONE.

[21] Taylor A.M., Shih J., Ha G., Gao G.F., Zhang X., Berger A.C., Schumacher S.E., Wang C., Hu H., Liu J. (2018). Genomic and Functional Approaches to Understanding Cancer Aneuploidy. Cancer Cell.

[22] Kumar M., Bowers R.R., Delaney J.R. (2020). Single-cell analysis of copy-number alterations in serous ovarian cancer reveals substantial heterogeneity in both low- and high-grade tumors. Cell Cycle.

[23] Ciriello G., Miller M.L., Aksoy B.A., Senbabaoglu Y., Schultz N., Sander C. (2013). Emerging landscape of oncogenic signatures across human cancers. Nat. Genet..

[24] Suelmann B.B.M., Rademaker A., van Dooijeweert C., van der Wall E., van Diest P.J., Moelans C.B. (2022). Genomic copy number alterations as biomarkers for triple negative pregnancy-associated breast cancer. Cell. Oncol..

[25] The Cancer Genome Atlas Research Network (2011). Integrated genomic analyses of ovarian carcinoma. Nature.

[26] Cancer Genome Atlas Research Network (2012). Comprehensive genomic characterization of squamous cell lung cancers. Nature.

[27] Wang S., Li H., Song M., Tao Z., Wu T., He Z., Zhao X., Wu K., Liu X.-S. (2021). Copy number signature analysis tool and its application in prostate cancer reveals distinct mutational processes and clinical outcomes. PLoS Genet..

[28] Meijer G.A., Hermsen M.A., Baak J.P., van Diest P.J., Meuwissen S.G., Beliën J.A., Hoovers J.M., Joenje H., Snijders P.J., Walboomers J.M. (1998). Progression from colorectal adenoma to carcinoma is associated with non-random chromosomal gains as detected by comparative genomic hybridisation. J. Clin. Pathol..

[29] Tang J., Le S., Sun L., Yan X., Zhang M., Macleod J., Leroy B., Northrup N., Ellis A., Yeatman T.J. (2010). Copy number abnormalities in sporadic canine colorectal cancers. Genome Res..

[30] Han X., Tan Q., Yang S., Li J., Xu J., Hao X., Hu X., Xing P., Liu Y., Lin L. (2019). Comprehensive Profiling of Gene Copy Number Alterations Predicts Patient Prognosis in Resected Stages I-III Lung Adenocarcinoma. Front. Oncol..

[31] Driver J., Hoffman S.E., Tavakol S., Woodward E., Maury E.A., Bhave V., Greenwald N.F., Nassiri F., Aldape K., Zadeh G. (2022). A molecularly integrated grade for meningioma. Neuro Oncol..

[32] Hieronymus H., Murali R., Tin A., Yadav K., Abida W., Moller H., Berney D., Scher H., Carver B., Scardino P. (2018). Tumor copy number alteration burden is a pan-cancer prognostic factor associated with recurrence and death. eLife.

[33] Thomas R., Fiegler H., Ostrander E.A., Galibert F., Carter N.P., Breen M. (2003). A canine cancer-gene microarray for CGH analysis of tumors. Cytogenet. Genome Res..

[34] Mochizuki H., Shapiro S.G., Breen M. (2016). Detection of Copy Number Imbalance in Canine Urothelial Carcinoma With Droplet Digital Polymerase Chain Reaction. Vet. Pathol..

[35] Li J., Liang J., Xu Y., Tan W., Chen G., Ou T. (2025). Droplet digital PCR assay for precise determination of FRS2 gene copy number in bladder cancer. BMC Cancer.

[36] Wu S., Ou T., Xing N., Lu J., Wan S., Wang C., Zhang X., Yang F., Huang Y., Cai Z. (2019). Whole-genome sequencing identifies ADGRG6 enhancer mutations and FRS2 duplications as angiogenesis-related drivers in bladder cancer. Nat. Commun..

[37] Chen Y., McGee J., Chen X., Doman T.N., Gong X., Zhang Y., Hamm N., Ma X., Higgs R.E., Bhagwat S.V. (2014). Identification of druggable cancer driver genes amplified across TCGA datasets. PLoS ONE.

[38] Spasova V., Mladenov B., Rangelov S., Hammoudeh Z., Nesheva D., Serbezov D., Staneva R., Hadjidekova S., Ganev M., Balabanski L. (2021). Clinical impact of copy number variation changes in bladder cancer samples. Exp. Ther. Med..

[39] Hughesman C.B., Lu X.J.D., Liu K.Y.P., Zhu Y., Towle R.M., Haynes C., Poh C.F. (2017). Detection of clinically relevant copy number alterations in oral cancer progression using multiplexed droplet digital PCR. Sci. Rep..

[40] Ramos-Rodriguez A.J., McFadden J.R., Momtahen S., LeBlanc R.E., Yan S., Chaudhari A.S., Cloutier J.M., Stevanovic M., Barney R., Syku M. (2024). A novel method to assess copy number variations in melanocytic neoplasms: Droplet digital PCR for precise quantitation of MYC and MYB genes. J. Cutan. Pathol..

