# Supplementary material for: Copy Number Alterations in Canine Urothelial Carcinomas: The Impact of Tumour Purity
Source: Vet Sci. 2026 May 8;13(5):459. doi: 10.3390/vetsci13050459 (PMC13211511; doi:10.3390/vetsci13050459)
Supplement: Supplementary file 1 [file vetsci-13-00459-s001.zip › Table S1.pdf]

Table S1: Signalment data for each of the 76 canine urothelial carcinomas and the 24 non-neoplastic bladder samples. Owner-reported breed, sex (F, female; M, male), neutering status (N, neutered; I, intact), age at the time of sampling, BRAF V595E mutation test results, CNA results, tissue and tumour measurements, tumour morphology included in the study. CNA-positive if both normalized ratios (CFA13/CFA19 and CFA36/CFA19) exceeded the predefined ratio × correction factor threshold of 1.23. Sorted in alphabetical order of the breeds. CNA-positive samples are highlighted in light grey.
n/a = data not available; pos., positive; neg., negative; CFA, canine chromosome; CNA, copy number analysis.

UC samples (*n* = 76)

case ID	breed	sex	neutering status (N=neutered, I=intact)	age (years)	BRAF^{V595E} mutation 1: pos. 0: neg.	CNA 1: pos. 0: neg.	CFA13/19	CFA36/19	total tissue area (mm²)	total tumour tissue area (mm²)	tumour proportion	tumour morphology
1	American Cocker Spaniel	M	N	12	0	1	6.26	2.74	90	53	58%	mixed
2	Basset Hound	F	N	11	1	1	1.91	3.04	173	74	43%	mixed
3	Beagle	M	N	13	1	1	5.40	1.54	8	4	46%	classic urothelial
4	Border Collie	F	N	11	1	1	2.58	2.58	11	3	28%	solid
5	Border Collie	F	N	12	1	1	1.71	2.12	147	33	23%	classic urothelial
6	Border Collie	F	N	14	1	1	4.78	3.41	71	26	37%	classic urothelial
7	Cavalier King Charles Spaniel	F	N	7	1	1	1.98	1.63	34	4	11%	classic urothelial
8	Cavalier King Charles Spaniel	M	N	7	1	0	4.61	0.93	9	3	37%	mixed
9	Cavalier King Charles Spaniel	F	N	7	1	1	3.65	1.31	3	1	48%	classic urothelial
10	Cavalier King Charles Spaniel	F	n/a	8	1	1	1.60	2.40	41	4	10%	mixed
11	Cavalier King Charles Spaniel	M	N	11	1	0	1.58	1.06	372	6	2%	mixed
12	Cocker Spaniel	F	N	9	1	1	3.24	4.20	494	403	82%	classic urothelial
13	Cocker Spaniel	F	N	11	1	0	3.01	0.97	7	5	61%	classic urothelial
14	Cocker Spaniel	M	N	11	1	1	2.01	1.74	106	12	11%	classic urothelial
15	Cocker Spaniel	F	N	14	1	0	1.30	0.84	13	6	51%	classic urothelial
16	Collie	F	I	14	1	1	1.50	1.72	452	131	29%	mixed
17	Crossbreed	F	N	8	0	1	1.52	1.48	8	1	10%	mixed
18	Crossbreed	F	N	8	1	1	2.88	1.58	3	1	49%	mixed
19	Crossbreed	F	n/a	8	1	1	2.02	1.32	12	3	24%	classic urothelial
20	Crossbreed	M	N	9	1	1	1.94	1.37	7	3	47%	cystic

21	Crossbreed	M	N	9	1	0	1.86	1.19	590	55	9%	classic urothelial
22	Crossbreed	F	n/a	9	1	1	2.77	3.39	255	25	10%	microinvasive
23	Crossbreed	F	N	10	1	1	2.24	1.68	276	44	16%	classic urothelial
24	Crossbreed	F	N	11	1	1	1.92	1.86	5	2	35%	mixed
25	Crossbreed	M	n/a	11	1	1	2.95	2.04	366	127	35%	mixed
26	Crossbreed	M	N	11	1	1	1.63	1.38	41	6	15%	mixed
27	Crossbreed	F	N	12	1	0	1.96	1.15	95	6	6%	classic urothelial
28	Crossbreed	F	N	13	1	1	2.98	1.84	165	25	15%	mixed
29	Crossbreed	F	N	13	1	1	1.60	2.16	48	40	83%	classic urothelial
30	Crossbreed	F	N	13	1	1	2.79	1.80	333	109	33%	classic urothelial
31	Crossbreed	M	N	13	1	0	1.89	1.04	311	97	31%	mixed
32	Crossbreed	F	N	13	0	0	0.77	1.14	401	17	4%	solid
33	Crossbreed	F	N	n/a	0	0	0.66	1.17	302	23	8%	mixed
34	Dachshund	F	N	10	0	0	0.72	1.11	486	64	13%	mixed
35	Dachshund	M	N	11	1	1	2.81	1.71	28	9	32%	classic urothelial
36	Dachshund	M	N	12	1	1	2.47	3.52	381	236	62%	classic urothelial
37	Elkhound	F	n/a	8	1	1	4.03	2.60	11	4	40%	mixed
38	English Cocker Spaniel	F	N	8	1	1	3.21	1.95	6	3	51%	mixed
39	Fox Terrier	M	N	6	1	0	2.59	1.05	102	5	5%	classic urothelial
40	Fox Terrier	F	N	11	1	1	1.98	1.43	6	3	46%	solid
41	Jack Russell Terrier	F	n/a	9	1	1	2.78	1.72	372	60	16%	classic urothelial
42	Jack Russell Terrier	M	N	11	1	1	2.19	2.91	260	193	74%	classic urothelial
43	Jack Russell Terrier	F	N	11	1	1	2.33	1.30	344	106	31%	classic urothelial
44	Jack Russell Terrier	M	N	n/a	1	1	1.95	1.41	62	28	45%	microinvasive
45	Jack Russell Terrier	M	I	n/a	1	0	1.81	1.09	195	33	17%	mixed
46	Labrador Retriever	M	N	8	1	1	2.48	1.34	41	8	19%	squamous
47	Labrador Retriever	M	N	9	1	1	1.69	2.52	28	0.03	0.1%	classic urothelial
48	Labrador Retriever	M	n/a	9	1	1	6.91	4.09	2	1	29%	classic urothelial
49	Labrador Retriever	M	N	9	1	0	1.79	1.10	26	8	32%	mixed
50	Labrador Retriever	M	N	9	1	1	2.13	1.62	24	12	52%	cribriform
51	Labrador Retriever	F	N	10	1	1	1.51	2.42	7	1	16%	classic urothelial
52	Labrador Retriever	M	n/a	11	1	1	2.50	1.84	417	181	44%	mixed
53	Labrador Retriever	F	N	12	0	0	0.63	1.14	16	1	8%	glandular, nested
54	Labrador Retriever	F	N	12	1	0	1.96	1.05	12	8	67%	mixed

55	Labrador Retriever	F	N	13	1	0	1.19	2.20	16	5	31%	mixed
56	Magyar Vizsla	F	N	10	1	1	5.33	2.08	19	4	24%	mixed
57	Pointer	M	n/a	11	1	1	2.75	1.27	305	53	17%	classic urothelial
58	Samoyed	F	N	13	1	1	2.04	1.66	28	13	48%	classic urothelial
59	Scottish Terrier	M	N	9	1	1	2.77	1.76	247	106	43%	classic urothelial
60	Scottish Terrier	F	N	10	1	1	2.85	2.86	142	125	88%	classic urothelial
61	Scottish Terrier	F	N	10	1	1	3.07	1.54	273	65	24%	mixed
62	Shetland Sheepdog (Sheltie)	F	n/a	8	1	1	1.86	1.36	67	11	16%	classic urothelial
63	Shetland Sheepdog (Sheltie)	F	N	9	1	1	3.26	5.21	9	2	18%	classic urothelial
64	Shetland Sheepdog (Sheltie)	F	n/a	9	1	0	1.66	1.02	111	35	31%	mixed
65	Staffordshire Bull Terrier	F	N	8	1	1	3.27	2.96	13	5	37%	classic urothelial
66	Tibetan Terrier	M	N	10	1	0	2.11	1.15	22	2	11%	glandular, nested
67	Weimaraner	F	N	9	1	1	2.07	1.29	14	7	47%	mixed
68	Weimaraner	M	I	10	1	1	1.44	1.49	42	3	6%	mixed
69	Welsh Corgi	M	N	11	1	1	1.56	1.26	100	76	76%	cribriform
70	Welsh Springer Spaniel	F	N	8	0	1	3.49	1.85	99	74	75%	classic urothelial
71	West Highland White Terrier	F	N	7	1	1	4.13	1.51	8	3	35%	classic urothelial
72	West Highland White Terrier	F	N	9	1	1	2.98	1.74	52	18	36%	classic urothelial
73	West Highland White Terrier	M	N	9	1	1	2.36	1.62	312	86	27%	mixed
74	West Highland White Terrier	F	n/a	9	1	1	3.20	1.69	265	133	50%	classic urothelial
75	West Highland White Terrier	F	N	11	1	1	2.68	1.86	313	65	21%	classic urothelial
76	West Highland White Terrier	M	N	12	1	1	2.39	1.89	432	91	21%	classic urothelial

Non-neoplastic bladder samples (*n* = 24)

case ID	breed	sex	neutering status (N=neutered, I=intact)	age (years)	BRAF^{V595E} mutation 1: pos. 0: neg.	CNA 1: pos. 0: neg.	CFA13/19	CFA36/19	histomorphological diagnosis
77	Border Collie	M	N	5	0	0	0.90	0.99	cystitis
78	Border Terrier	M	N	10	0	0	0.80	1.05	polyp
79	Boxer	M	I	8	0	0	0.84	1.03	polyp
80	Crossbreed	M	N	1	0	0	0.70	1.06	cystitis

81	Crossbreed	M	I	1	0	0	0.97	1.04	cystitis
82	Crossbreed	M	N	5	0	0	0.92	0.92	cystitis
83	Crossbreed	M	N	6	0	0	0.73	0.96	polyp
84	Crossbreed	F	N	7	0	0	0.92	1.06	cystitis
85	Crossbreed	F	N	9	0	0	1.03	1.01	cystitis
86	Crossbreed	F	N	9	0	0	0.80	0.94	cystitis
87	Crossbreed	M	N	9	0	0	0.69	0.81	polyp
88	Crossbreed	F	N	10	0	0	0.72	0.90	cystitis
89	Crossbreed	n/a	n/a	n/a	0	0	0.93	1.00	cystitis
90	Dalmatian	F	N	7	0	0	0.80	1.24	cystitis
91	Dobermann	M	N	3	0	0	0.57	0.88	cystitis
92	English Cocker Spaniel	M	N	5	0	0	0.70	0.99	polyp
93	Foxterrier	F	N	12	0	0	0.70	0.90	polyp
94	French bulldog	F	N	10	0	0	0.82	0.99	polyp
95	Great Dane	M	I	5	0	0	0.94	1.01	cystitis
96	Miniature Pinscher	M	I	12	0	0	0.86	0.97	polyp
97	Pug	F	N	11	0	0	1.16	1.28	cystitis
98	Shih Tzu	F	N	8	0	0	0.85	1.42	cystitis
99	Tibet Terrier	F	I	5	0	0	0.61	1.01	cystitis
100	n/a	M	I	12	0	0	0.95	1.07	cystitis
