# Supplementary figures and images for: Copy Number Alterations in Canine Urothelial Carcinomas: The Impact of Tumour Purity
Source: Vet Sci. 2026 May 8;13(5):459. doi: 10.3390/vetsci13050459 (PMC13211511; doi:10.3390/vetsci13050459)

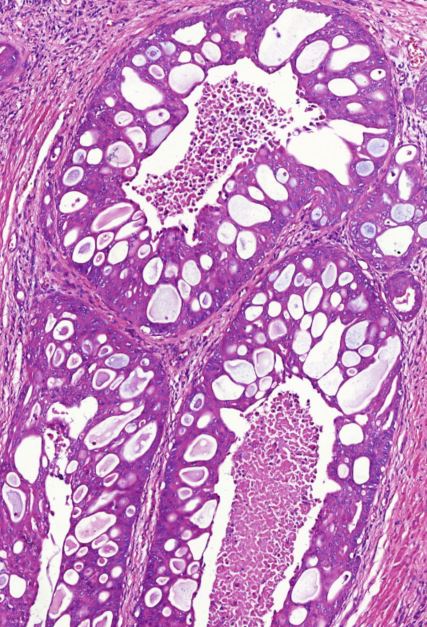

Supplement: Supplementary file 1 [file vetsci-13-00459-s001.zip › vetsci-4291442-original-images/Figure_1a.PNG]

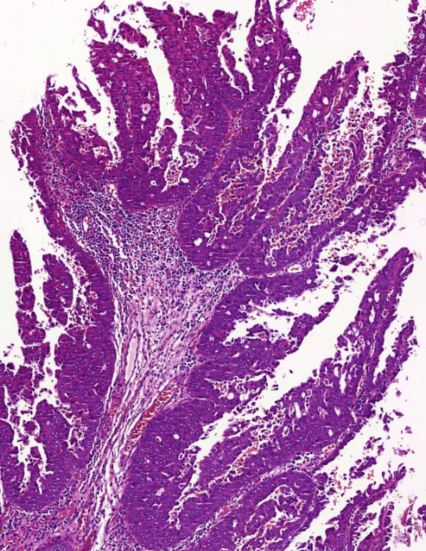

Supplement: Supplementary file 1 [file vetsci-13-00459-s001.zip › vetsci-4291442-original-images/Figure_2a.PNG]
